# The effect of thermo-oxidative ageing on crystallization, dynamic and static mechanical properties of long glass fibre-reinforced polyamide 10T composites

**DOI:** 10.1098/rsos.172029

**Published:** 2018-06-20

**Authors:** Jian Wang, Lingtong Li, Yong He, Haishuo Song, Xiaolang Chen, Jianbing Guo

**Affiliations:** 1 Department of Polymer Material and Engineering, College of Materials and Metallurgy, Guizhou University, Guiyang 550025, People's Republic of China; 2 Key Laboratory of Advanced Materials Technology Ministry of Education, School of Materials Science and Engineering, Southwest Jiaotong University, Chengdu 610031, People's Republic of China; 3 National Engineering Research Center for Compounding and Modification of Polymer Materials, Guiyang, Guizhou 550014, People's Republic of China

**Keywords:** composites, thermo-oxidative ageing, performances, morphology

## Abstract

The performances and microstructure of long glass fibre-reinforced polyamide 10T (PA10T/LGF) composites that experienced different ageing temperatures (160 and 200°C) with increasing ageing time are characterized by differential scanning calorimetry (DSC), mechanical analysis, thermogravimetric analysis (TGA) and scanning electron microscopy to probe the correlation between properties of the composites and thermo-oxidative ageing. The DSC results show that PA10T/LGF composites occur on degradation, the fracture of molecular chains and the destruction of crystallization structure, which leads to the crystallization and melting peaks of PA10T/LGF composites to shift to high temperature. On the basis of dynamic mechanical analysis data, the reduction of the interfacial bonding between the glass fibre and PA10T matrix and the motion of molecular chain segments result in the thermo-oxidative ageing of composites. According to the calculation of activation energy (*E*), thermo-oxidative temperature and ageing time can bring about the decline of the *E* value, proving the deterioration in performance of PA10T/LGF composites. In view of TGA, the increase in the thermo-oxidative temperature and ageing time promotes the degradation of PA10T/LGF composites. The tensile, flexural and notched impact strengths of PA10T/LGF composites decline with prolonging the ageing temperature and time. The surface of materials produces some microcracks and the cross-section surface of PA10T/LGF composites becomes rougher.

## 1. Introduction

Polyamide (PA), an important engineering thermoplastic, is composed of a large quantity of repeated amide groups in the molecular chains. PA has many advantages such as excellent mechanical performance, low frictional coefficient, fine chemical resistance, satisfying electric properties, abrasion resistance and thermal stability, and draws a lot of attention as potential material [1–3]. By virtue of the outstanding mechanical properties, PA has been widely applied in electronics, household appliances, and the automobile and aviation industries [4]. A great deal of research, in recent years, has focused on enhancing the heat resistance and moisture absorption of PA for long-term application prospects. Some researchers [5,6] reported that the physiochemical properties of PA and suitability for specific applications are largely dependent on the structure of the main chains. The structure of PA has direct impact on its moisture absorption, particularly for thermal stability. The main chain is divided into an aliphatic chain and an aromatic chain [7]. The glass fibre reinforces the PA to improve behaviour [8–10]. Poly(decamethylene terephthalamide) (PA10T) prepared from the homopolymerization of terephthalic acid and decamethylene diamine is a new type of heat-resistant PA [11]. Because the aromatic rings are grafted to the PA10T molecular backbone, PA10T embraces much better thermal stability than other aliphatic PAs. At the same time, methylene is linked with the PA10T closely and the polymer chain can endow the matrix with melting machinability and flexibility. In addition, the moisture absorption and dimensional stability of PA10T are lower than those of other nylon materials, such as PA6 and PA66, which can improve the strength and stiffness of traditional nylon material owing to the decline of the water absorption. PA10T can not only maintain the strength, high rigidity and high size stability, but also improve other properties, thus the PA10T materials can be applied to a wide range of fields. Consequently, glass fibre-reinforced PA composites are increasingly important engineering materials due to their high level of mechanical performances and temperature resistance [12–14].

The currently accepted definition of PA composites is that they are susceptible to radiation, heat, light, moisture, UV, microorganisms, and so on, and it is the sum of all possible specific interactions between the durability and applications of composites, excluding storage, machining and transportation. While this is conceptually straight-forward, it is composed of several interacting factors, including thermo-oxidative ageing, the integrated effect of oxygen and heat, both permanent and induced, which play an important role in the ageing process of the polymer materials, leading to the auto-oxidative scission of the main chain. Thereupon, it is worth studying the relationship between the thermo-oxidative effect and the properties of the composites. It is both a physical and a chemical phenomenon. Further to this, heat can accelerate oxidation, resulting in the formation of peroxide, which in turn breaks main chains at high temperature [15–17]. The addition of antioxidants, including phenols and amine, has been examined for the ability to avoid those defects of polymer composites. For example, the tensile properties of the PA6/glass fibre composites [18,19] were enhanced compared with those of the pure samples.

Until now, a lot of composites with brittle strength and segmental motion have been developed for large-scale industrial application. Given the results of some literature [18,20,21], the work described the static and dynamic mechanical properties of long glass fibre (LGF)-reinforced PA6 composites which were exposed at 160°C for a long time. It was found that the glass transition temperature (*T*_g_) of the composites shifted to a higher temperature with increasing the ageing time, which was attributed to the cross-linking reactions of the molecular chain. In accordance with the earlier studies [22,23], the ageing properties of the short glass fibre-reinforced PA composites changed due to the interface bonding between glass fibre and PA matrix. Nevertheless, the acquaintance on the properties of heat-resisting PA such as PA10T under the process of thermo-oxidative ageing was small.

The aim of this study is to investigate the property evolution of LGF-reinforced PA10T composites that are exposed to 160 and 200°C. We determine the thermo-oxidative ageing behaviour and the mechanical properties of LGF/PA10T by several diverse ageing periods from 10 to 50 days to predict the optimal conditions for the application of LGF/PA10T composites. Additionally, direct measurements of LGF/PA10T composites at different environments are performed to elucidate the thermo-oxidative ageing process of LGF/PA10T composites.

## 2. Experimental

### 2.1. Material and method

Poly(decamethylene terephthalamide) (PA10T) (commercial grade Vicnyl 600T) was purchased from Kingfa Science and Technology (China). The density and water absorption ratio of the product are 1.12 g cm^−3^ and 1.15%, respectively. The consequent glass fibres (commercial grade ECT4301H) were prepared by Chongqing International Composite Materials Co., Ltd., China, with an average diameter of 17 µm.

### 2.2. Preparation of long glass fibre-reinforced polyamide10T composites

**Figure 1.**
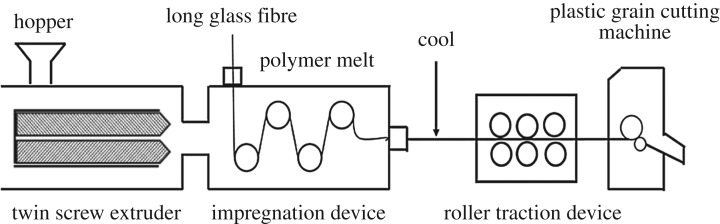
The melt-impregnation process for LGF/PA10T composites.

Figure 1 illustrates the preparation process of PA10T/LGF composites. The samples were prepared by a twin-screw extruder according to a previous procedure [24]. PA10T was dried at approximately 100°C for 8 h prior to processing. The PA10T/LGF composites were prepared by melt blending in a co-rotating twin-screw extruder (*L*/*D* = 40, *D* = 40 mm, Coperion Keya Machinery Nanjing, China), and the ramping temperature profile range from hopper to die was 285, 290, 295, 305, 310 and 315°C. The glass fibre, pulled into the impregnation device, was an essential process using a number of rollers. When the glass fibre was cool, the successive extrudates were cut into pellets at a length of about 10 mm by a plastic grain cutting machine. The speed of the bob was 16.5 r.p.m. The weight fraction of LGF was about 40%. The screw speed was 175 r.p.m., and the relative screw speed of the feeding was 12.5 r.p.m. The traction speed of the glass fibre roving was 4 m min^−1^, and the impregnation temperature was 330 to 340°C. The melt-impregnation equipment was also used to add LGF to interact with the PA10T matrix. The product was dried at 80°C for 24 h, and then injection-moulded (Type CJ80M3 V, Chen De Plastics Machinery Guangdong, China) into different samples for testing and characterization at a barrel temperature of 320°C.

To evaluate the effects of thermo-oxidative ageing on the performances of LGF/PA10T composites, it was necessary to take measurements to accelerate the ageing process of composites. The composite specimens were aged in a ventilated oven at 160 and 200°C for 0, 10, 20, 30, 40 and 50 days. The aged samples were moved at regular time intervals and stored at 25 ± 3°C for at least 24 h prior to testing.

### 2.3. Measurement and characterizations

#### 2.3.1. Mechanical property test

The tensile strength and flexural strength were performed by a WDW-10C universal testing machine, in accordance with ASTM D-638 and ASTM D-790, respectively. The testing speeds of the tensile and flexural strength were set at 50 and 2 mm min^−1^, respectively. According to the ASTM D-256 standard, a SANS ZBC-4B impact tester equipped with a pendulum of 2.75 J was used to explore the Notched Izod impact strength. All experiments were carried out at 25°C, and all statistical data were recorded including the average of the tests.

#### 2.3.2. Differential scanning calorimetry

Differential scanning calorimetry (DSC) analysis was carried out by using a Q10 (TA Instruments, USA) thermal analyser to evaluate the non-isothermal crystallization and melting behaviour of the composites under a nitrogen atmosphere. About 8 mg of sample was placed in Al-pans. The sample was then heated from room temperature to 320°C and held for 5 min at this temperature, and the previous thermal history of the composites was eliminated completely. It was cooled naturally to 150°C at a constant rate of 10°C min^−1^, and then heated to 320°C at the same heating rate again.

#### 2.3.3. Dynamic mechanical analysis

Dynamic mechanical analysis (DMA) was conducted on a Q800 analyser (TA Instruments). The imposed frequencies were 1, 5, 10, 15 and 20 Hz, and the amplitude was 1.5 µm in the bending mould. The specimens (60 × 12.7×3.18 mm) were heated from room temperature to 300°C at a heating rate of 2°C min^−1^.

#### 2.3.4. Thermogravimetric analysis

The thermal properties of the composites were performed by thermogravimetric analysis (TGA) (TA, Q-50 instruments, Co., Ltd., USA) under a nitrogen atmosphere. The scan range was from room temperature to 700°C with a heating rate of 10°C min^−1^.

#### 2.3.5. Scanning electron microscopy

Scanning electron microscopy (SEM) images, used for probing on the surfaces and fracture cross-sections of both aged and unaged LGF/PA10T specimens, were received on a KYKY-2800 (KYKY Technology Development, China) instrument at an accelerating voltage of 10 kV under high vacuum, after gold coating surface treating.

## 3. Results and discussion

### 3.1. Differential scanning calorimeter

**Figure 2.**
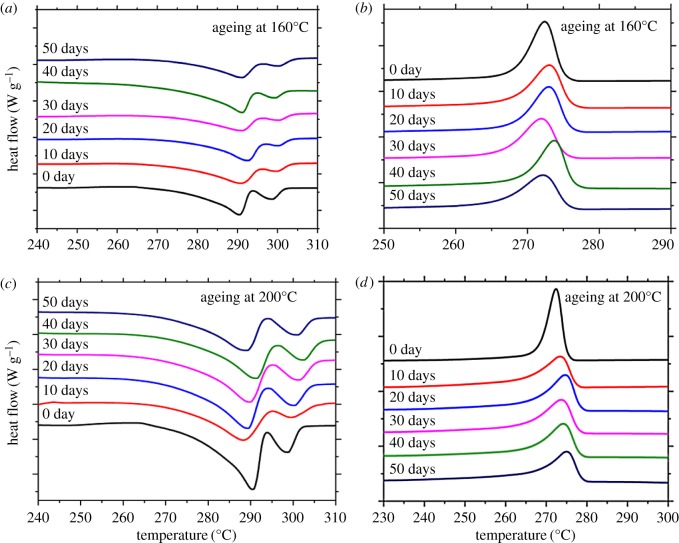
DSC curves of PA10T/LGF composites at different thermo-oxidative ageing temperature and time: (*a*), (*c*) the melting curves; (*b*), (*d*) the crystallization curves.

Varieties of LGF/PA10T exposed to thermal ageing at 160 and 200°C, in DSC thermograms, are shown in figure 2, and the detailed data are listed in table 1. The influence of thermo-oxidative degradation can be proved by the changes in melting and crystallization behaviours. The crystallization peak temperature (*T*_c_), onset crystallization temperature (*T*_onset_), melting peak temperature (*T*_m_) and melting enthalpy (Δ*H*_m_) vary with the changes in the ageing time and ageing temperature. From table 1, when the thermo-oxidative temperature is 160°C, *T*_c_ changes little and *T*_onset_ slightly declines with increasing ageing time, but the change in *T*_onset_ is not apparent at the beginning. However, *T*_c_ and *T*_onset_ values of the composites increase a little with prolonged ageing time. It is notable that *T*_c_ and *T*_onset_ of the composites at 200°C are enhanced slightly compared with those of the composites at 160°C, which indicates that the ageing temperature has an effect on the crystallization behaviours of the composites. In view of the above phenomenon, the crystallization structure of the PA10T/LGF composites is likely to be changed because the long-time thermo-oxidative effect leads to worse destruction of molecular chains and more products of microcracks. When PA10T suffers from heat and oxygen, the weak area of the molecular chains in the amorphous region fractures, leading to the formation of micromolecules. On the one hand, micromolecules will lead to the reduction of the crystallization peak in the PA10T; on the other hand, micromolecules, applied to be the nucleating agent, can induce the form of the new spherocrystal and the shape of integrated crystallization, and the samples should be placed at a higher crystallization temperature and longer crystallization process [25]. It is of importance that the poorly integrated molecular chain impairs the ability of crystallization of PA10T in the PA10T/LGF composites. Compared with the specimens at 160°C, the samples at 200°C are confronted with more severe ageing processes, leading to worse breakage of the molecular chain and sharper reduction in molecular weight. At higher temperatures, the fracture of the molecule chain leads to motion of molecules.

**Table 1.** The crystallization and melting parameters of PA10T/LGF composites after thermo-oxidative ageing at 160 and 200°C.

	ageing at 160°C					ageing at 200°C				
ageing time (days)	*T*_onset_ (°C)	*T*_c_ (°C)	*T*_m1_ (°C)	*T*_m2_ (°C)	Δ*H*_m_ (J g^−1^)	*T*_onset_ (°C)	*T*_c_ (°C)	*T*_m1_ (°C)	*T*_m2_ (°C)	Δ*H*_m_ (J g^−1^)
0	275.7	272.4	290.5	298.6	26.6	275.7	272.4	290.5	298.6	26.6
10	276.1	273.0	290.9	299.9	25.7	277.4	273.4	288.3	299.6	21.1
20	275.9	272.9	292.5	300.2	23.5	278.4	274.6	289.5	300.4	23.4
30	275.2	271.9	291.2	300.4	22.2	278.0	273.6	289.9	301.5	22.8
40	276.5	273.8	291.9	301.9	23.9	278.5	274.1	291.5	302.5	21.8
50	275.6	272.1	291.3	300.4	22.3	278.7	275.0	289.2	301.2	18.7

At the same time, the melting enthalpy at the certain functional temperature reduces in contrast with that of the unaged composites. However, with the increasing ageing time, the melting enthalpy of the composites at 200°C clearly decreases compared with the composites at 160°C. In view of this phenomenon, thermo-oxidative ageing temperature and time play an important role in the melting process of composites. On the one hand, the fracture of molecular in the amorphous can weaken the entanglement of the molecular chain, and the reduction of crystallization is also a vital factor for the decrease of △*H*_m._ On the other hand, the decrease in the degree of crystallinity has a negative impact on the properties of composites during the degradation of the LA10T/LGF composites. Thereby the change in the crystallization is consistent with the variation of the Δ*H*_m_. The decline of the Δ*H*_m_ indicates that the crystallization structure of the PA10T/LGF composites is destroyed during the thermo-oxidative process.

As shown in figure 2, the DSC curves are good indicators of performances and properties of PA10T/LGF composites. From the curves, it is reasonable to state that the incorporation of LGF into PA10T happens to be the variation of crystallization and melting, which will bring about the degradation of the composites, change of the crystal thickness and formation of the carbon layer. According to figure 2, it is clear that unaged and aged PA10T/LGF composites appear to be double melting peaks [26,27] due to a lower cooling rate, inducing secondary crystallization after the first crystallization. The main reasons for this are as follows: a portion of crystals form more complete and thicker wafers, and others produce more irregular and smaller wafers during the melt-quenching process. At the same time, the degradation of molecular chains also causes the formation of small molecules, and small molecules are prone to form perfect wafers, inducing the form of double-melting peaks. In addition, because the samples should be annealed before melting or cooling slowly, the samples have been fully crystallized, leading to what appears to be a crystallization peak. Meanwhile, the formation of a char layer covers the surface of the PA10T/LGF composites along with the ageing time and the ageing temperature, and the break of the molecular chain results in the above phenomenon.

### 3.2. Dynamic mechanical analysis

**Figure 3.**
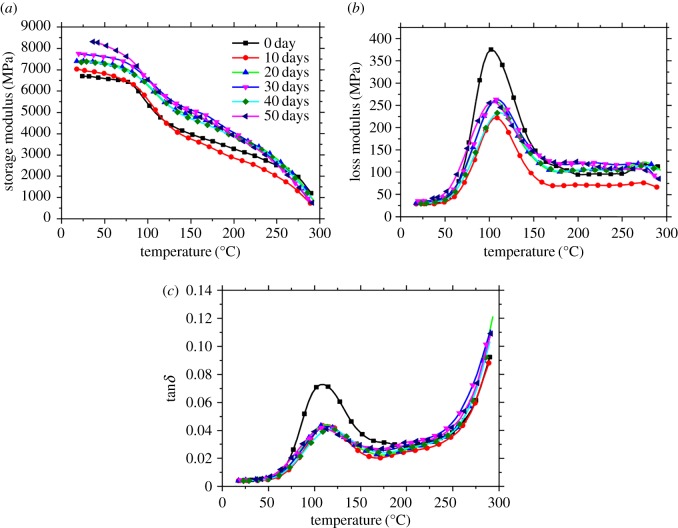
Storage modulus (*a*), loss modulus (*b*) and tan*δ* (*c*) of PA10T/LGF composites with different ageing time at 160°C.

**Figure 4.**
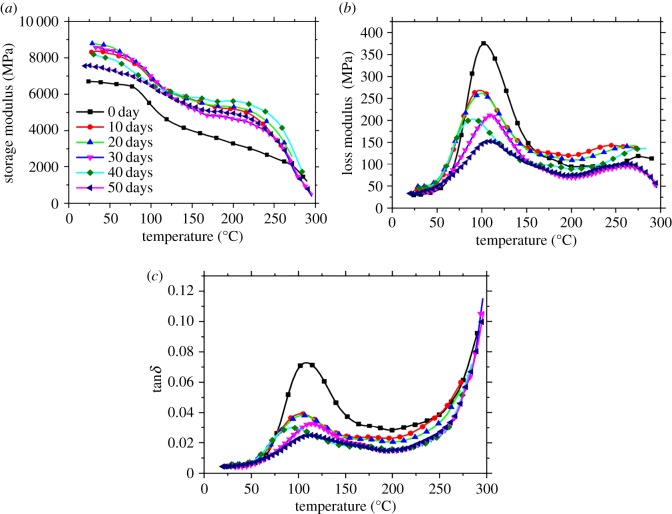
Storage modulus (*a*), loss modulus (*b*) and tan*δ* (*c*) of PA10T/LGF composites with different ageing time at 200°C.

Figures 3 and 4 show the storage modulus (*E*′), loss modulus (*E*^″^) and tan*δ* of PA10T/LGF composites with different ageing time at 160 and 200°C. The interactions between mechanical features and molecular chains in the amorphous phase are measured by DMA curves to calculate the compatibility between the glass fibre and the matrix PA10T [28,29]. As shown in figures 3 and 4, the storage modulus of samples aged at 160 and 200°C is evidently promoted in comparison with the unaged samples. When it comes to elevate ageing time, the integrated region causes grievous destruction, invoking the gap existing in molecule and molecule. As a result of the easier movement of micromolecule and molecular segment, the molecular chains gradually turn into flexible chains. When the temperature is lower than that of the *T*_g_, molecule chains are frozen and barely engaged in molecule movement. Thus, the initiated curves are nearly flat straight lines. Surpassing *T*_g_, molecule chains become flexible and molecule chain shifts slightly. At the beginning of any curve, it may be deduced that the glass transition platform grows wider with increasing thermo-oxidative temperature from 160 to 200°C. With enhancing temperature, the glass state, glass transition and rubbery state of PA10T/LGF composites appear. As shown in figures 3 and 4, the change of temperature leads to the decrease of *E*′. The unaged sample shows a relatively high initial *E*′ value, and the reason is that in the glass state, the frozen molecular chain segments of PA10T/LGF composites hinder the movement of the molecular chain and only some small groups can move, exhibiting a high rigidity; the mass of glass fibres is randomly distributed in the PA10T matrix to form a rigid network, thereby increasing the rigidity of PA10T/LGF composite materials; the polarity of the PA10T can also promote the rigidity of the PA10T/LGF composites. But, in the glass transition process, the *E*′ decreases rapidly because of the motion of the polymer chain segment. In the rubbery state, the PA10T/LGF composites exhibit a rubber-like state and the *E*′ remains relatively stable. Additionally, it is difficult to observe the glass transition process, especially when the ageing time exceeds 30 days, and there is almost no change for the *E*′ value. At the same time, the glass transition disappears, which is attributed to the formation of mass of oxidized carbides on the specimen surface owing to the degradation and deterioration of the composites.

For the *E*^″^ values of PA10T/LGF composites in figures 3 and 4, it is apparent that the peak value of *E*^″^ is highest about 100°C and the loss modulus of the unaged samples is higher than that of the aged samples, regardless of 160 and 200°C ageing. At the same oxidative time, the value of the loss modulus tends to decrease gradually, while the thermo-oxidative temperature changes from 160 to 200°C. This phenomenon is attributed to the variation of the viscosity of the composites. By view of the increase of thermo-oxidative temperature, the motor ability of the molecule gradually increases and the viscosity decreases, leading to the decrease in *E*^″^. Maybe the intermolecular forces recede as the result of the change of temperature and when the reduction of *E*^″^ is remarkable.

The tan *δ* curves are susceptible to the variations of the molecular structure during the process of ageing. In general, considering the corresponding temperature of tan*δ* peak as *T*_g_ is a widespread fact, and the molecular chains at *T*_g_ are easier to move with the largest energy loss and hysteresis phenomenon. It is observed from figure 3 that the peak of tan *δ* of the composites at 160°C shifts to higher temperature compared with unaged samples, indicating that *T*_g_ promotes as a consequence of cross-linking between the PA10T matrix and the LGF. In addition, the motion of molecular chain segments also plays a significant role in improving *T*_g_. Along with increasing the thermo-oxidative time, the degradation of the composites and the fracture of molecular chains reduce the interfacial bonding between glass fibre and PA10T matrix and internal friction weakens, obtaining the result that the loss modulus of the multiple system gradually drops.

### 3.3. Calculation of activation energy

The apparent activation energy (*E*) is beneficial to elucidate vividly the thermo-oxidative behaviour of the composites when the composites undergo the glass transition process. The value of *E* stands for the relationship between mobility and time scale and represents the energy barrier of glass transition relaxation [30]. *T*_g_ value can reflect the relation between mobility of polymer chains and temperature. Obviously, the DMA tests are completed over a temperature range of −125 to 125°C and at five frequencies (1, 5, 10, 15 and 20 Hz).

According to the classic Arrhenius equation, molecular relaxation time (s) may be expressed as follows:

3.1 $$\tau =\tau_{0}{\text{e}}^{\Delta E-\gamma \sigma /RT},$$

where Δ*E* and *σ* are the *E* of the relaxation process and the stress, respectively, *γ* is the variable, *R* is the gas constant and *T* is the absolute temperature. Here, *τ*_0_ is a hypothetical relaxation time at infinite temperature.

Here, the stress (*σ*) is small, so the following simplified version is used.

3.2 $$\tau =\tau_{0}\;{\text{e}}^{\Delta E/RT}$$

and

3.3 $$\ln\tau =\ln\tau_{0}+\frac{\Delta E}{RT}.$$

The relaxation times were obtained from the relationship

3.4 $$\tau =\frac{1}{\omega }.$$

A combination of equations (3.3) and (3.4) leads to

3.5 $$\begin{array}{rl} \ln\left(\frac{1}{\omega }\right) & =\ln\tau_{0}+\frac{\Delta E}{RT}, \end{array}$$

3.6 $$\begin{array}{rl} \omega & =2\pi f \end{array}$$

3.7 $$\begin{array}{rl} \;\text{and}\;\ln(2\pi f) & =-\ln\tau_{0}-\frac{\Delta E}{RT}. \end{array}$$

If we plug *τ* = 1/2πf_0_ into the equation (3.7), we will get the following equation:

3.8 $$\begin{array}{rl} \ln(2\pi f) & =-\ln\frac{1}{2\pi f_{0}}-\frac{\Delta E}{RT}, \end{array}$$

3.9 $$\begin{array}{rl} \ln(2\pi f)+\ln\frac{1}{2\pi f_{0}} & =-\frac{\Delta E}{RT}, \end{array}$$

3.10 $$\begin{array}{rl} \ln\left(\frac{2\pi f}{2\pi f_{0}}\right) & =-\frac{\Delta E}{RT} \end{array}$$

3.11 $$\begin{array}{rl} \;\text{and}\;\ln\left(\frac{f}{f_{0}}\right) & =-\frac{\Delta E}{RT}. \end{array}$$

**Figure 5.**
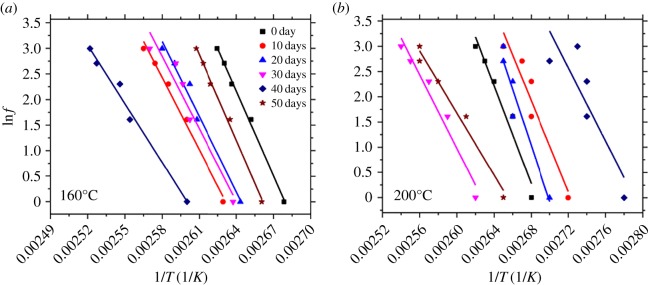
Arrhenius plots of relaxation times versus 1/*T* and the respective linear fits of PA10T/LGF composites at 160°C (*a*) and 200°C (*b*).

The Arrhenius equation has the following form:

3.12 $$f=f_{0}\exp\left(\frac{-\Delta E}{RT}\right),$$

where *f* and *f_0_* are the best frequencies and a constant, respectively. Figure 5 shows the linear relationship between ln*f*_max_ against the reciprocal of 1/*T*.

According to equation (3.12), a plot of ln*f* versus 1/*T* should give a straight line with a slope that is proportional to the *E* (energy barrier to motion) associated with the *α* elaxation process of the PA10T/LGF composites [31]. It is observed from table 2 that the *E* value gradually declines with the thermo-oxidative temperature at the same ageing time. In fact, *E* is defined as the energy required to convert the molecule from the normal state to the active state where the chemical reaction is likely to occur. *E* can be used to represent the minimum energy required for the occurrence of the chemical reaction [32]. In other words, the lower the *E*, the easier the reaction. In view of this, the oxidative process of the PA10T/LGF composites is prone to occur when the thermo-oxidative temperature ranges from 160 to 200°C, which is attributed to the reduction of the interface bonding and the enhancement of the small molecule motion. To some extent, relatively high temperature can break the macromolecules into the micromolecules, and the micromolecules move acutely and collide with each other, resulting in the easy occurrence of the reaction. In addition, the polymer changes from rigidity to flexibility due to the decrease of the viscosity at relatively high temperature. Furthermore, the *E* value decreases little by little with increasing ageing time at the thermo-oxidative temperature. The reason is that the number of the molecules that can react is increasing, and interface bonding between the LGF and the PA10T is gradually decreased with enhancing ageing time. On the whole, the thermo-oxidative temperature and the ageing time can affect the *E* value, resulting in the degradation and deterioration of the PA10T/LGF composites.

**Table 2.** The activation energy of the PA10T/LGF composites at 160 and 200°C.

activation energy (*E *kJ^−1^ mol^−1^)	0 day	10 days	20 days	30 days	40 days	50 days
160°C	458.3	391.9	406.8	392.4	318.1	463.8
200°C	429.7	402.0	397.6	305.4	320.4	259.5

### 3.4. Thermal stability

**Figure 6.**
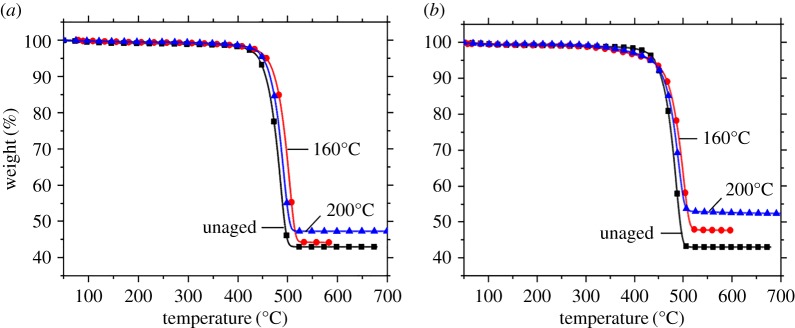
The TGA curves of PA10T/LGF composites with different thermo-oxidative ageing time at different ageing temperature: (*a*) 10 days; (*b*) 50 days.

TGA, a highly efficient method, is used to probe the degradation and thermal stability of composites by studying the weight loss of the material during the thermo-oxidative process [33]. Figure 6 shows the TGA curves of PA10T/LGF composites at different thermo-oxidative temperatures under a heat flow of nitrogen at a heating rate 10°C/min. It is found from figure 6 that the charred residues gradually increase with increasing thermo-oxidative temperature compared with the unaged samples. Although the tendency to increase is not obvious, a slight variation manifests the change of the composites. However, the PA10T/LGF composites show remarkable enhancement of charred residues with the thermo-oxidative temperature at 200°C in comparison with the unaged composites when the PA10T/LGF composites undergo 50 day thermo-oxidative process. By increasing the ageing temperature, the PA10T/LGF composites gradually bring about the degradation of matrix and form a higher degree of carbonization, showing a relatively high thermal stability. The glass fibre and the carbon layers formed on the surface of the composites during thermo-oxidative ageing can effectively prevent the fabrication of inorganic additives and carriage of volatiles, and thus reduce the heat release speed during the thermal decomposition process, inducing increasing charred residues of polymer with enhancing the ageing temperature. Therefore, temperature is a critical factor for PA10T/LGF composites and degradation and carbonization of the substrate occupy a more serious position at a relatively high temperature (200°C) that corresponds to the dramatic decrease in mechanical properties.

**Figure 7.**
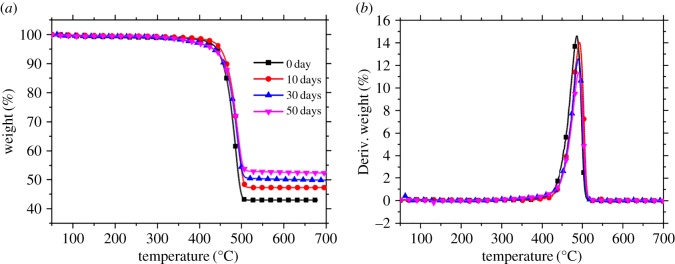
The TGA curve (*a*) and DTG curve (*b*) of PA10T/LGF composites with different thermo-oxidative ageing time at 200°C.

Figure 7 shows the TGA and DTG curves of PA10T/LGF composites with different thermo-oxidative ageing time at 200°C, and the corresponding data are listed in table 3. The variation of *T*_5%_ (initial decomposition temperature) is trivial with the increase of ageing time from 0 to 50 days, showing relatively high thermal stability. The reason is that glass fibres and carbon layers formed on the surface of the composites during thermo-oxidative ageing can effectively hinder the volatilization of small molecules, and thus lead to the decrease in heat release speed during the thermal decomposition process. However, the *T*_5%_ values of the samples that undergo the thermo-oxidative process at 200°C slightly increase and then decline, and the charred residues increase from 43.0% of the unaged samples to 53.1% in the samples aged 50 days. After thermo-oxidative ageing at 200°C, the TGA curves of samples become more and more gentle when the ageing time is gradually increased as shown in figure 7. The speed of the weight loss clearly declines with increasing ageing time, especially when the ageing time exceeds 30 days, leading to an extremely low weight and likely to result in a straight line, showing that masses of matrix transform into carbon owing to the long ageing period. In view of the above phenomenon, it is speculated that the structure of the samples is damaged due to the degradation of the samples and the carbon layers at the surface of the samples are involved, which can hinder the thermal degradation of the materials. Therefore, it is concluded that thermo-oxidative ageing under a high temperature or with increasing thermo-oxidation will accelerate the formation of carbonization and the occurrence of the degradation in PA0T/LGF composites, thus bringing about deterioration in the whole performance.

**Table 3.** TGA data of PA10T/LGF composites after thermo-oxidative ageing at 200°C.

ageing time (days)	*T*_5%_ (°C)	*T*_max_ (°C)	residues at 600°C (%)
0	441.0	486.9	43.0
10	450.5	493.1	47.4
30	432.8	489.3	50.4
50	433.1	491.1	53.1

### 3.5. Mechanical properties

**Figure 8.**
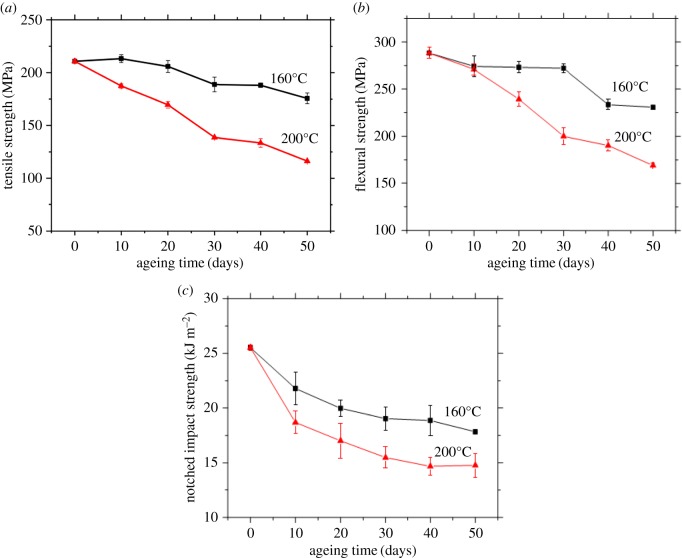
Mechanical properties of PA10T/LGF composites as a function of ageing time at different thermo-oxidative ageing temperature: (*a*) tensile strength; (*b*) flexural strength; (*c*) notched impact strength.

Figure 8 shows the variations of tensile strength, flexible strength and notched impact strength of PA10T/LGF composites with various thermo-oxidative ageing at 160 and 200°C. The tensile strength, flexural strength and notched impact strength of PA10T/LGF composites gradually decline and the trend of reduction becomes gentle with increasing the ageing time. In addition, the mechanical properties of PA10T/LGF composites aged at 200°C decrease more remarkably than those of PA10T/LGF composites aged at 160°C. After 50 days thermo-oxidative ageing at 160°C, the tensile strength, flexural strength and notched impact strength of PA10T/LGF composites decrease to 175.7 MPa, 230.8 MPa, 17.8 kJ m^−2^, respectively. However, after thermo-oxidative ageing at 200°C for 50 days, the tensile strength, flexural strength and notched impact strength of PA10T/LGF composites decrease to 116.3 MPa, 169.1 MPa and 14.8 kJ m^−2^, respectively. From the above results, it is concluded that the mechanical properties of PA10T/LGF composites at 200°C decrease obviously compared with that at 160°C. Obviously, the mechanical properties of the composites are impaired with increasing ageing temperature, which is attributed to the degradation of the composites, the decrease in intermolecular interaction and the reduction in molecular weight. When the composites suffer from severe thermo-oxidative ageing at higher temperature, the degradation of matrix gives rise to a break in the molecular chains and destruction of molecular arrangement and accelerates the formation of small molecules, rendering the decrease in intermolecular interaction between PA10T and LGF. Based on the effect of thermo-oxidative ageing, the mechanical properties of PA10T/LGF composites are impaired.

With the destruction of the crystalline structure of PA10T/LGF composites and the decline in the crystalline [34], the mechanical properties of PA10T/LGF composites deteriorate. Generally, fracture of the molecular chains forms low-molecular-weight products and leads to the formation of cross-links during thermo-oxidative ageing. At the same time, oxidation of the composites plays an inevitable and important role on the polymer matrix, which results in the decline of the properties of materials because of the decrease in interface binding force between the LGF and the PA10T matrix. By comparing the curves of the composites at 160°C and 200°C in figure 8, it is observed that the thermo-oxidative ageing of the matrix and the deterioration of interface bonding have a more serious and severe impact on the composites at 200°C. Thus, the mechanical properties of the composites after ageing at 200°C, including tensile, flexural and notched impact strengths, decline seriously.

### 3.6. Morphology observation

**Figure 9.**
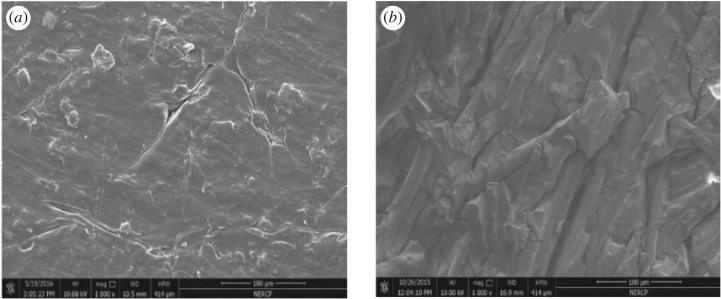
Morphologies of unaged and aged PA10T/LGF composites: (*a*) unaged sample; (*b*) aged 50 days sample.

The surface micrographs of the PA10T/LGF composites after 0 and 50 days thermo-oxidative ageing at 200°C are shown in figure 9. It is clearly seen from figure 9 that the surface of the samples after 50 days of thermo-oxidative ageing becomes rougher than that of the unaged sample. This is explained because the matrix layers that cover the initiative glass fibre surfaces are damaged, and a large number of glass fibres are directly exposed to the air, leading to the formation of the crack. In view of the variation of the surface of the samples with enhancing ageing time, thermo-oxidative action results in severe breakage of the PA10T molecular chains and brings about debonding between the glass fibre and the matrix, inducing the formation of small cracks. With the growth of tiny cracks, oxygen infiltrates the gap of the crack, enlarging the contact area between the matrix and the oxygen [35], and it is inevitable that the factors further accelerate the ageing process of the matrix. From figure 9 the pit and the delicate cracks easily constitute cracks and reduce the energy such that the break in the resin matrix is one of the main reasons for the decline in the static mechanical properties. Furthermore, the surfaces of material suffering from the thermo-oxidative effect undergo oxidative degradation and rupture of the molecular chains, and then expand the inside matrix, so the mechanical properties of the material finally are destroyed.

**Figure 10.**
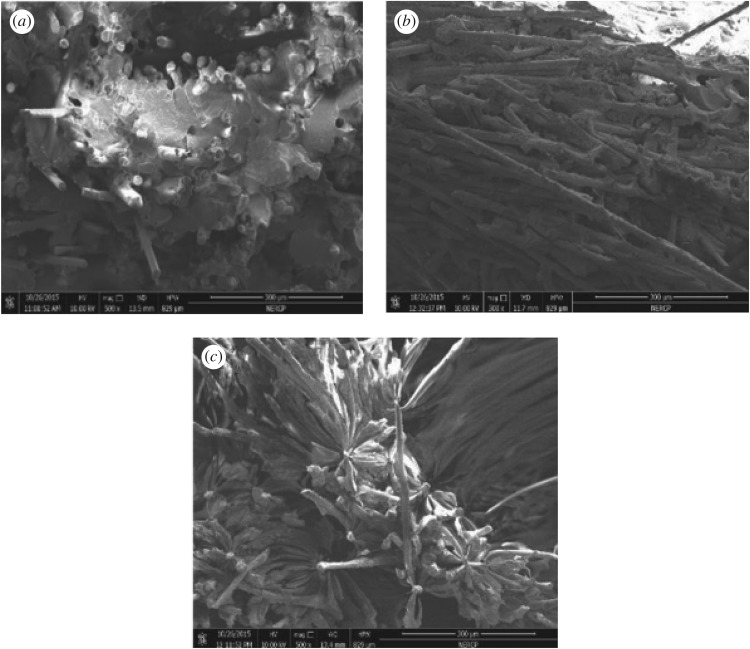
SEM images for unaged and aged impact fracture surfaces of PA10T/LGF composites: (*a*) unaged sample; (*b*) aged 30 days sample; (*c*) aged 50 days sample.

The impact fracture surface micrographs of PA10T/LGF composites after 0, 30 and 50 days thermo-oxidative ageing at 200°C are shown in figure 10. The cross-section surface of PA10T/LGF composites turns out to be rougher than that of the unaged sample. For unaged composites, the glass fibres are well covered by PA10T matrix. By prolonging the ageing time, the surfaces of materials produce some microcracks which act as Griffin flaws and crack initiation sites. At the same time, the enhanced thermal embrittlement of the composites and the oxidative scission of PA10T molecular chains cause the molecular chain to split into fragments. With increasing ageing time, the interface area between the matrix and the glass fibre is cut down because of the severe degradation of the substrate and formation of microcracks and debris [36], thus aggravating the mechanical properties. Overall, the microcracks lead to the formation of the stress concentration point and the growth of the cracks, resulting in the mechanical properties declining to a large extent.

## 4. Conclusion

The LGF-reinforced PA10T composites are prepared by the melt extrusion method, and injection splines are subjected to thermo-oxidative ageing with different exposure times (160 and 200°C). The effects of thermo-oxidative ageing on thermal stability, crystallization, mechanical properties and morphology of PA10T/LGF composites are investigated using DSC, TGA, DMA and TGA, etc. Based on the experimental results, it is found that the decreased molecular weight, the interfacial bedonding between the glass fibre and the matrix, and the carbonization of PA10T are the crucial factors that result in the deterioration of the properties and the degradation of the composites. The results from DSC show that the melting temperature range of PA10T/LGF composites is broadened with prolonging the thermos-oxidative time because the degradation of the composites gives rise to the formation of small molecular. The variation of the *T*_g_ reflects the cross-linking of the composites with changes in the ageing time. Using SEM cracks and matrix fragments can be seen on the surface of the composites with increasing ageing time and ageing temperature. After long-term ageing, the interface interaction between the LGF and PA10T matrix declines due to the interface debonding of the composites, which results in the reduction of the mechanical properties of the composites. Besides, the effect of thermo-oxidative ageing on *T*_5%_ and *T*_max_ of the composites is evaluated by TGA curves. It is concluded that the thermo-oxidative ageing temperature and time are the crucial factors of material degradation and deterioration, which determines the application time of materials to a large extent.

## Data accessibility

This manuscript does not have extra data. All data are provided in full in the results section of this paper.
